# Supplementary material for: Association of mean corpuscular volume with 28-day mortality in sepsis patients: A retrospective cohort study using eICU data
Source: PLoS One. 2025 Apr 21;20(4):e0321213. doi: 10.1371/journal.pone.0321213 (PMC12011257; doi:10.1371/journal.pone.0321213)
Supplement: S1 Table — (DOCX) [file pone.0321213.s001.docx]

**S1 Table.** Univariate analysis for 28day mortality

| Characteristic | Statistics | ICU 28day mortality | *P*-value |
| --- | --- | --- | --- |
| Gender |  |  |  |
| Male | 4622 (49.10%) | 1 |  |
| Female | 4792 (50.90%) | 1.08 (0.94, 1.24) | 0.2723 |
| Age (years) | 65.76 ± 15.86 | 1.01 (1.01, 1.02) | <0.0001 |
| BMI | 29.02 ± 9.13 | 0.99 (0.98, 1.00) | 0.0075 |
| Respiratory rate (bpm) | 30.55 ± 14.50 | 1.02 (1.01, 1.02) | <0.0001 |
| Heart rate (/min) | 114.01 ± 28.62 | 1.01 (1.00, 1.01) | <0.0001 |
| MAP (mmHg) | 76.82 ± 43.83 | 1.00 (1.00, 1.00) | 0.0388 |
| Acute Physiology Score III | 60.37 ± 24.31 | 1.03 (1.02, 1.03) | <0.0001 |
| Apache IV score | 73.96 ± 25.49 | 1.03 (1.02, 1.03) | <0.0001 |
| AIDS |  |  |  |
| No | 9268 (99.69%) | 1 |  |
| Yes | 29 (0.31%) | 3.06 (1.30, 7.19) | 0.0101 |
| Hepatic failure |  |  |  |
| No | 9102 (97.90%) | 1 |  |
| Yes | 195 (2.10%) | 3.25 (2.33, 4.54) | <0.0001 |
| Metastatic cancer |  |  |  |
| No | 9008 (96.89%) | 1 |  |
| Yes | 289 (3.11%) | 1.91 (1.39, 2.63) | <0.0001 |
| Albumin(g/dL) | 2.47 ± 0.61 | 0.61 (0.53, 0.70) | <0.0001 |
| Lactate(mmol/L) | 2.52 ± 2.21 | 1.16 (1.13, 1.19) | <0.0001 |
| Platelets (cells x 109/L) | 202.24 ± 114.89 | 1.00 (1.00, 1.00) | <0.0001 |
| Hemoglobin (g/dL) | 10.34 ± 2.13 | 0.96 (0.93, 0.99) | 0.0143 |
| RDW(%) | 16.13 ± 2.62 | 1.13 (1.10, 1.16) | <0.0001 |
| White blood cell count (cells x 109/L) | 15.59 ± 11.78 | 1.01 (1.00, 1.01) | 0.0354 |

BMI, body mass index; MAP,mean arterial pressure;RDW ,red cell distribution width;MCHC, mean cellular hemoglobin concentration;AIDS,acquired immunodeficiency syndrome.
