# Supplementary material for: Association of mean corpuscular volume with 28-day mortality in sepsis patients: A retrospective cohort study using eICU data
Source: PLoS One. 2025 Apr 21;20(4):e0321213. doi: 10.1371/journal.pone.0321213 (PMC12011257; doi:10.1371/journal.pone.0321213)
Supplement: S2 Table — (DOCX) [file pone.0321213.s002.docx]

**S2 Table. Relationship between MCV and 28-day mortality during ICU stay less than 48 hours**

| **Outcomes** | **Crude Model** | |  | **Model Ⅰ** | |  | **Model Ⅱ** | |
| --- | --- | --- | --- | --- | --- | --- | --- | --- |
|  | **OR(95%CI)** | ***P*-value** |  | **OR(95%CI)** | ***P*-value** |  | **OR(95%CI)** | ***P*-value** |
| MCV(fl)quartile |  |  |  |  |  |  |  |  |
| Q1 | Reference |  |  | Reference |  |  | Reference |  |
| Q2 | 1.15 (0.91, 1.44) | 0.241 |  | 1.10 (0.87, 1.39) | 0.421 |  | 1.11 (0.76, 1.63) | 0.58 |
| Q3 | 1.38 (1.10, 1.72) | <0.001 |  | 1.25 (1.00, 1.57) | 0.05 |  | 1.13 (0.77, 1.65) | 0.523 |
| Q4 | 2.10 (1.70, 2.58) | <0.001 |  | 1.91 (1.55, 2.36) | <0.001 |  | 1.27 (0.89, 1.82) | 0.186 |
| MCV(fl)quartilecontinuous | 1.05 (1.04, 1.06) | <0.001 |  | 1.04 (1.03, 1.05) | <0.001 |  | 1.01 (0.99, 1.03) | 0.31 |

Crude model: we did not adjust other covariants; Model Ⅰ adjusted for: Age and Gender; Model Ⅱ adjusted for: Age, Gender, BMI, Temperature, Respiratory rate, Heart rate, MAP, Acute Physiology Score III, APACHE IV score, AIDS, Hepatic failure, Metastatic cancer, Immunosuppression, Albumin, Lactate, Platelets, Hemoglobin, RDW and White blood cell count.
