# Supplementary material for: Association of mean corpuscular volume with 28-day mortality in sepsis patients: A retrospective cohort study using eICU data
Source: PLoS One. 2025 Apr 21;20(4):e0321213. doi: 10.1371/journal.pone.0321213 (PMC12011257; doi:10.1371/journal.pone.0321213)
Supplement: S3 Table — (DOCX) [file pone.0321213.s003.docx]

| **Outcomes** | **Crude Model** | |  | **Model Ⅰ** | |  | **Model Ⅱ** | |
| --- | --- | --- | --- | --- | --- | --- | --- | --- |
|  | **OR(95%CI)** | ***P*-value** |  | **OR(95%CI)** | ***P*-value** |  | **OR(95%CI)** | ***P*-value** |
| MCV(fl)quartile |  |  |  |  |  |  |  |  |
| Q1 | Reference |  |  | Reference |  |  | Reference |  |
| Q2 | 1.04 (0.85, 1.28) | 0.705 |  | 1.02 (0.81, 1.23) | 0.996 |  | 1.07 (0.85, 1.35) | 0.583 |
| Q3 | 1.07 (0.87, 1.31) | 0.521 |  | 1.03 (0.81, 1.22) | 0.966 |  | 1.01 (0.77, 1.22) | 0.792 |
| Q4 | 1.41 (1.16, 1.71) | <0.001 |  | 1.31 (1.07, 1.59) | 0.007 |  | 1.19 (0.95, 1.49) | 0.138 |
| MCV(fl)quartilecontinuous | 1.02 (1.01, 1.03) | <0.001 |  | 1.02 (1.01, 1.03) | <0.001 |  | 1.01 (1.00, 1.02) | 0.05 |
|  |  |  |  |  |  |  |  |  |

**S3 Table. Relationship between MCV and 28-day mortality when adjusted for sepsis subtypes**

Crude model: we did not adjust other covariants; Model Ⅰ adjusted for: Age and Gender; Model Ⅱ adjusted for: Age, Gender, BMI, Temperature, Respiratory rate, Heart rate, MAP, Acute Physiology Score III, APACHE IV score, Shock, AIDS, Hepatic failure, Metastatic cancer, Immunosuppression, Albumin, Lactate, Platelets, Hemoglobin, RDW and White blood cell count.
