# Supplementary material for: Association of mean corpuscular volume with 28-day mortality in sepsis patients: A retrospective cohort study using eICU data
Source: PLoS One. 2025 Apr 21;20(4):e0321213. doi: 10.1371/journal.pone.0321213 (PMC12011257; doi:10.1371/journal.pone.0321213)
Supplement: Attachment — Submitted filename: review paper comments.docx [file pone.0321213.s004.docx]

**Topic: Association of mean corpuscular volume with 28-day mortality in sepsis patients: A retrospective cohort study using eICU data**

**Allover strengths**

- The study investigates the association between mean corpuscular volume (MCV) and 28-day mortality in sepsis patients, a crucial clinical issue.
- The study applies **multivariable regression models**, **smoothed curve fitting**, and **threshold effects analysis** to examine the dose-response relationship.
- Identifying **MCV as a prognostic marker** could aid in early risk assessment and clinical decision-making in sepsis management.

**General comment**

- The manuscript presents **a well-conducted study with clinically relevant findings**, supported by **strong statistical methods and a large dataset**.
- However, **some methodological and conceptual clarifications** are needed to strengthen the study’s impact and **improve its translational relevance for clinicians**.
- Your reference needs to be the standard and updated. Most of them are below 2022.
- Some sentences are **overly complex or contain grammatical errors** (e.g., “The extant evidence indicates…” → should be “The existing evidence indicates…”). I suggest a final **proofreading** or **language revision** to enhance readability.

**Abstract**

- Please include the period for data collection.

**Introduction**

1. The introduction discusses prior studies on MCV and mortality in **chronic diseases (e.g., kidney failure, cardiovascular disease)** but does not explain **why MCV might be relevant in sepsis**. I am not clinically competent to suggest; however, it is better to expand on the **pathophysiological mechanisms** linking MCV changes to sepsis outcomes.
2. In line 41: the author put the reference in a recommended way “Modifications in MCV have been linked to unfavorable prognoses in a multitude of pathological conditions [2, 3, 4, 5, 6, 7]. “ please revise based on [2 -7].
3. Excellent introduction about the burden. However, you have to write from the global to local, if possible in your study areas' findings. “In the United States, approximately 750,000 cases of sepsis occur annually, resulting in around 215,000 deaths, with a mortality rate of 28.7%[9, 10]. Globally, approximately 1,400 individuals perish daily as a result of sepsis, with an estimated mortality rate of 20%” I suggest you to put first global them USA,,

Methods and materials

1. At lines 69-71; the author said “The database comprises vital clinical data from over 200,000 patients admitted to 335 ICUs across 20870 hospitals in the United States during the period from 2014 to 2015” . It's a good study, however, I am afraid the current practice may have a different scenario. I prefer to review the recent data.
2. At this in my view, I suggest data quality issues like pretest, data quality control and soon. Also stating ethical clearance with an approved reference number is advisable.
3. The study **excludes** patients with ICU stays **less than 48 hours**. However, early deaths (within 48 hours) might be informative for the relationship between MCV and sepsis outcomes. I suggest to **Justify the exclusion of early ICU discharges or deaths**, as this could introduce selection bias.

Result

1. At baseline characteristics, it will be nice to state some important variables, like age, Gender, BMI,,, and then the table will follow.
2. AT Table 1 APACHE and ICU were not stated on your table key note. Please include. As table standards, it needs to clearly describe which population data is displayed. So, please revise the heading of the table.
3. Although the study adjusts for multiple confounders, some **key factors are not explicitly addressed**: For example: **Use of blood transfusions** (which can alter MCV levels) and **Severity of sepsis subtypes** (e.g., septic shock vs. non-shock sepsis). I suggest acknowledging these limitations or considering a **sensitivity analysis** if data is available.

Discussion

1. I suggest writing the limitation of the study in the final paragraph of the discussion section.
2. The discussion mentions that **clinicians should monitor MCV**, but it is unclear how this should influence clinical practice. Address **whether MCV should be incorporated into sepsis risk stratification models** or **how it compares with other established biomarkers (e.g., lactate, SOFA score).**
