# Supplementary material for: Association of mean corpuscular volume with 28-day mortality in sepsis patients: A retrospective cohort study using eICU data
Source: PLoS One. 2025 Apr 21;20(4):e0321213. doi: 10.1371/journal.pone.0321213 (PMC12011257; doi:10.1371/journal.pone.0321213)
Supplement: Attachment — Submitted filename: Response to Additional Reviewer And Editor Comment.docx [file pone.0321213.s005.docx]

**Manuscript ID: PONE-D-24-53001**

**Title: Association of mean corpuscular volume with 28-day mortality in sepsis patients: A retrospective cohort study using eICU data**

**Part 1. Response to Editor Comment**

**Dear Editors,**

We sincerely appreciate the editors’ insightful comments and constructive suggestions. We have carefully addressed each point raised, and the revisions are detailed below.

### 1. ****This study reported a significant association between MCV and mortality. Add a paragraph about the reported sepsis in this retrospective study.****

**Revision:**
A new paragraph has been added to the **Discussion** section Line 243-251) to contextualize sepsis epidemiology and clinical characteristics in our cohort:

"In this retrospective cohort, sepsis was defined using the ICD code (A41.9) and included patients with systemic inflammatory response syndrome secondary to infection. The 28-day ICU mortality rate in our cohort (9.38%) was significantly lower than the global mortality rate^[19]^. This difference may reflect advances in earlier sepsis identification and standardized management programs, such as the Surviving Sepsis Campaign guidelines^[20]^. Our population consisted primarily of older adults (mean age: 65.7±15.8 years), consistent with the demographics of sepsis, where age is a recognized risk factor for adverse outcomes^[21]^. In addition, the inclusion of 335 intensive care unit patients from 208 hospitals in the United States enhances the generality of our findings across different critical care Settings."

### 2.  Line 42 (page 2/19) In Chinese ........ Add reference

**Revision:**
The statement on Line 57 (Page 3/22) now cites the relevant reference:

"In Chinese patients, a lower MCV (less than 94 fl) was associated with an elevated risk of mortality^[2]^."

**Added Reference:**
[2]	Saito H, Tanaka K, Iwasaki T, et al. Hematological parameters of anemia and prognosis of non-dialysis-dependent chronic kidney disease: the Fukushima CKD cohort study[J]. Clin Exp Nephrol, 2023,27(1):55-65. doi:10.1007/s10157-022-02282-1.

3. **The data collected during 2014-2015. Add the reasons**

**Revision:**
A justification has been added to the **Data Source** subsection (Line 90-95):

"The eICU Collaborative Research Database (eICU-CRD) comprises de-identified clinical data from the years 2014 to 2015, as this timeframe offers the most comprehensive and standardized dataset available at the time of analysis^[17]^. Subsequent updates to the database were not publicly accessible during the study design phase. The uniformity of data collection protocols across intensive care units during these years ensures minimal variability in variable definitions and measurement practices."

4. **In the table 1, some results are not normally distributed. Therefore mean +/- SD . It is preferable to be median (Q1-Q3) as it was mentioned in the statistical section**

**Revision:**
All non-normally distributed variables in **Table 1** have been updated to **median (interquartile range)**. For example:

MAP (mmHg); Platelets (cells x 109/L); White blood cell count (cells x 109/L); Lactate(mmol/L)

A footnote has been added: "Continuous variables are presented as mean ± SD or median (IQR) based on distribution; categorical variables as n (%)."

5. **Rephrase the sentence in the line 174-176 to be obvious and clear because this paragraph is the fundamental of this study**

**Original:**
"A significant increase in the 28-day risk of death was observed with each 1 fl increase at MCV<83 fl (OR 1.10, 95% CI 1.02, 1.17, P=0.004). The significant positive correlation was absent when MCV was greater than 83 fl (OR 1.02, 95% CI 0.99, 1.02, P=0.31)."

**Revised ( Line 238 – 242 ):**
"The key finding revealed below the MCV threshold of 83 fl, each 1 fl increase was associated with a 10% rise in 28-day mortality risk (OR 1.10, 95% CI 1.02–1.17, P=0.004). Above 83 fl, no significant association was observed (OR 1.01, 95% CI 0.99–1.02, P=0.31), indicating a nonlinear relationship with diminishing effects at higher MCV levels."

**Additional Revisions:**

1.Minor grammatical edits and formatting adjustments applied throughout.

2.Updated references to ensure consistency with journal guidelines.

We believe these revisions address all concerns raised and significantly strengthen the manuscript. Thank you for your consideration.

****Part 2.** Response to Reviewer Comments**

**Dear Reviewers,**

We express our sincere gratitude for the insightful feedback provided by the reviewers. We have meticulously addressed each comment to enhance the manuscript's clarity, scientific rigor, and translational relevance. Below, we present a detailed, point-by-point response to the reviewers' concerns. ****1. Strengths of the Study****

- ****Reviewer’s Comment:****

**The study investigates the association between MCV and 28-day mortality in sepsis patients, a crucial clinical issue. The study applies multivariable regression models,** smoothed curve fitting, and threshold effects analysis to examine the dose-response relationship. Identifying MCV as a prognostic marker could aid in early risk assessment and clinical decision-making in sepsis management.”

**Response:**
We thank the reviewer for recognizing the strengths of our study. We agree that MCV holds potential as a prognostic marker in sepsis, and our findings emphasize its clinical utility for risk stratification.

#### 2. ****General Comments****

#### ****Reviewer’s Comment:**** The manuscript presents a well-conducted study with clinically relevant findings, supported by strong statistical methods and a large dataset. However, some methodological and conceptual clarifications are needed to strengthen the study’s impact and improve its translational relevance for clinicians.

**Response:**
We appreciate the reviewer’s positive assessment of our work. Below, we address the specific methodological and conceptual concerns raised:

##### ****a. Methodological Clarifications****

****Data Source and Timeframe:**
We clarified the rationale for using the eICU-CRD database (2014–2015) in the Materials and Methods section. This timeframe was selected due to its standardized data collection protocols and completeness (Lines 90-95 ).**

1. ****Exclusion Criteria:**
   Added explicit justification for excluding patients with ICU stays <48 hours to ensure sufficient observation time for MCV assessment (Lines 110-114).**
2. ****Sensitivity Analyses:**
   Expanded descriptions of sensitivity analyses (e.g., follow-up <48 hours, adjustment for sepsis subtypes) to demonstrate robustness (Lines 156-159).**

****b. Conceptual Relevance****

****Clinical Implications:**
Enhanced the Discussion to emphasize how MCV integrates with existing biomarkers ( lactate, SOFA scores) for risk stratification in resource-limited settings (Lines 272-283).**

****Mechanistic Insights:**
Added a paragraph linking MCV to erythrocyte dysfunction and oxidative stress in sepsis pathophysiology (Lines 73-77).**

- ****Reviewer’s Comment:**
  Your reference needs to be the standard and updated. Most of them are below 2022.**

****Response:**
We have updated 18 references (45% of total citations) to include literature published between 2022 and 2024. For example:**

**Added recent studies on sepsis management (Ref. 4, 5, 6, 8, 19, 20).**

**Included 2024 publications on MCV-mortality relationships (Ref. 23, 33).**

- **Reviewer’s Comment:**
  Some sentences are overly complex or contain grammatical errors. I suggest a final proofreading or language revision to enhance readability.

**Response:**
We have thoroughly revised the manuscript for clarity and grammar. Key improvements include:

1. Simplified complex sentences (e.g., revised “The extant evidenced indicates…” to “Existing evidence indicates…” in Introduction).

2. Engaged a professional English editing service for final proofreading.

#### ****Abstract****

- **Reviewer’s Comment:**
  “Please include the period for data collection.”

**Response:**
We have added the data collection period (2014–2015) to the Abstract as requested.

**Revised Text (Lines 28-29):**
A retrospective cohort study was conducted using data from the eICU Collaborative Research Database from 2014–2015.

#### ****Introduction****

- **Reviewer’s Comment 1:**
  “The introduction discusses prior studies on MCV and mortality in chronic diseases (e.g., kidney failure, cardiovascular disease) but does not explain why MCV might be relevant in sepsis. Expand on the pathophysiological mechanisms linking MCV changes to sepsis outcomes.”

**Response:**
We have expanded the discussion of MCV’s relevance to sepsis by adding pathophysiological mechanisms.

**Revised Text (Lines73-76):**
Systemic inflammatory responses and oxidative stress in patients with sepsis may affect MCV by disrupting erythrocyte membrane stability or altering bone marrow hematopoietic function[12, 13]. Elevated MCV may reflect a state of chronic inflammation or nutrient deficiency that exacerbates organ dysfunction[13, 14].

- **Reviewer’s Comment 2:**

**In li**ne 41: the author put the reference in a recommended way “Modifications in MCV have been linked to unfavorable prognoses in a multitude of pathological conditions [2, 3, 4, 5, 6, 7]. “ please revise based on [2 -7].”

**Response:**
We apologize for the oversight .We have checked the format of the references and have revised the format of the references in the full text.

- **Reviewer’s Comment 3:**
  Excellent introduction about the burden. However, you have to write from the global to local, if possible in your study areas' findings. “In the United States, approximately 750,000 cases of sepsis occur annually, resulting in around 215,000 deaths, with a mortality rate of 28.7%[9, 10]. Globally, approximately 1,400 individuals perish daily as a result of sepsis, with an estimated mortality rate of 20%” I suggest you to put first global them USA,,

**Response:**
We have reordered the statistics to prioritize global data.

**Revised Text (Lines 65-67):**
Globally, approximately 1,400 individuals perish daily as a result of sepsis, with an estimated mortality rate of 20%^[7]^. In the United States, approximately 750,000 cases of sepsis occur annually, resulting in around 215,000 deaths, with a mortality rate of 28.7%^[8]^.

### ****Methods and Materials****

#### ****Reviewer’s Comment 1:****

#### At lines 69-71; the author said “The database comprises vital clinical data from over 200,000 patients admitted to 335 ICUs across 20870 hospitals in the United States during the period from 2014 to 2015” . It's a good study, however, I am afraid the current practice may have a different scenario. I prefer to review the recent data.

#### ****Response:**** We acknowledge the importance of using recent data. However, the eICU Collaborative Research Database (eICU-CRD) version available during our study design (publicly released in 2018) includes standardized data from 2014–2015. Subsequent updates were not accessible at the time of analysis. We have clarified this limitation in the revised manuscript.

#### ****Revised Text (Lines 90-95):**** The eICU Collaborative Research Database (eICU-CRD) comprises de-identified clinical data from the years 2014 to 2015, as this timeframe offers the most comprehensive and standardized dataset available at the time of analysis[17]. Subsequent updates to the database were not publicly accessible during the study design phase. The uniformity of data collection protocols across intensive care units during these years ensures minimal variability in variable definitions and measurement practices.

#### ****Reviewer’s Comment 2:****

#### At this in my view, I suggest data quality issues like pretest, data quality control and soon. Also stating ethical clearance with an approved reference number is advisable.

**Response:**
We have expanded the description of data quality control and explicitly stated the ethical approval reference.

****Revised Text (Lines 89, 97-104):****

The data is standardised cleaned and verified by the eICU-CRD team^[16]^.
The eICU-CRD team standardized, cleaned, and verified all clinical data to ensure quality^[15]^. After completing the course "Protection of Human Research Participants" (No. 65890571), the use of the database was approved by the Institutional Review Board (IRB) of Massachusetts Institute of Technology. Because of the retrospective nature of this study and the absence of direct patient intervention, the IRB of Massachusetts Institute of Technology waived the requirement for obtaining written informed consent. The study was compliant with the safe harbor provisions of the Health Insurance Portability and Accountability Act (HIPAA) and followed the Strengthening the Reporting of Observational Studies in Epidemiology (STROBE) statement.

- ****Reviewer’s Comment 3:****

The study excludes patients with ICU stays less than 48 hours. However, early deaths (within 48 hours) might be informative for the relationship between MCV and sepsis outcomes. I suggest to Justify the exclusion of early ICU discharges or deaths, as this could introduce selection bias.

**Response:**
We excluded patients with ICU stays <48 hours to minimize confounding from irreversible organ failure and transient MCV fluctuations. To validate this approach, we conducted a sensitivity analysis on this subgroup, which yielded results consistent with the primary analysis (S2 Table).

****Revised Text** (Lines157-158, 228–229):**

Initially, we examined follow-up outcomes in patients admitted to the ICU for less than 48 hours to determine the stability of core outcomes across varying time points.

We further investigated the association between MCV and mortality at a follow-up of less than 48 hours and obtained comparable results (S2 Table).

### ****Results****

- ****Reviewer’s Comment 1:****

At baseline characteristics, it will be nice to state some important variables, like age, Gender, BMI,,, and then the table will follow.

**Response:**
We have added expressions for the important demographic variables in Table 1.

**Revised Text (Lines 170-174):**

The demographic characteristics of the study population across MCV quartiles revealed that age increased progressively from 62.15 ± 16.52 years in Q1 to 67.99 ± 14.56 years in Q4 (p < 0.001), while BMI decreased from 29.68 ± 10.01 in Q1 to 28.32 ± 8.49 in Q4 (p < 0.001). The proportion of males and females did not differ significantly across quartiles (p = 0.128).

- ****Reviewer’s Comment 2:****

  AT Table 1 APACHE and ICU were not stated on your table key note. Please include. As table standards, it needs to clearly describe which population data is displayed. So, please revise the heading of the table.

**Response:**

We have revised the table heading and added missing abbreviations to the notes.

**Revised Text (Lines 176, 179-180):**

Table 1. Baseline characteristics of sepsis patients by MCV quartiles (N = 9,415).

APACHE IV: Acute Physiology and Chronic Health Evaluation IV; ICU: Intensive Care Unit.

- ****Reviewer’s Comment 3:****

Although the study adjusts for multiple confounders, some key factors are not explicitly addressed: For example: Use of blood transfusions (which can alter MCV levels) and Severity of sepsis subtypes (e.g., septic shock vs. non-shock sepsis). I suggest acknowledging these limitations or considering a sensitivity analysis if data is available.

****Response:****

**We sincerely appreciate the reviewer’s insightful critique regarding unaddressed confounders, specifically blood transfusions and sepsis subtypes.**

a. **Blood transfusions may transiently elevate MCV levels by introducing exogenous erythrocytes, potentially confounding the observed association between MCV and mortality. For instance, patients receiving transfusions might exhibit artificially higher MCV values unrelated to their underlying pathophysiology, leading to biased estimates. Regrettably, the eICU database does not include granular data on blood transfusion history (e.g., timing, volume, or type of transfusions). This limitation precludes direct adjustment for transfusion-related effects in our analysis.**

**We explicitly acknowledge this limitation in the revised Discussion (Lines 293–297):**

**In the current study, data on interventions in the initial stabilization phase were lacking; For example, blood transfusions may lead to elevated MCV levels and improved survival outcomes, a confounding variable that cannot be measured. Therefore, the conclusions of this study cannot be extrapolated to patients undergoing transfusion therapy.**

b. **The severity of sepsis subtypes (e.g., septic shock) may differentially influence both MCV levels and mortality risk. For example, septic shock patients often exhibit pronounced inflammation and microcirculatory dysfunction, which could independently alter erythrocyte indices and amplify mortality risks.**

To address this concern, we conducted a sensitivity analysis stratifying patients by sepsis subtypes (septic shock vs. non-shock) ( S3 Table). After adjusting for these subtypes, we found that the core results were consistent with the unadjusted results .**( Lines 229–231).**

### ****Discussion****

- ****Reviewer’s Comment1:****

I suggest writing the limitation of the study in the final paragraph of the discussion section.

**Response:**
The limitations section has been relocated to the final paragraph of the Discussion.

**Revised Text (Lines 285-308):**

**Study advantages and limitations**

**The present study has certain advantages. Firstly, it was a large-sample multicentre study; secondly, we analysed MCV as both a continuous and categorical variable; thirdly, we applied a two-segmented linear model to construct a threshold-effects analysis of the relationship between MCV and 28-day mortality in sepsis patients; and we used stratified analyses to avoid, as far as possible, the occurrence of chance in the statistical analyses and to improve the stability of the results.**

**The current study is not without its limitations. First, a common problem in observational studies is the presence of confounding factors that cannot be measured[35]. In the current study, data on interventions in the initial stabilization phase were lacking; For example, blood transfusions may lead to elevated MCV levels and improved survival outcomes, a confounding variable that cannot be measured. Therefore, the conclusions of this study cannot be extrapolated to patients undergoing transfusion therapy.**

**Second, the study was conducted over a 28-day period. During this time, we excluded patients who had been admitted to the ICU for less than 48 hours, as our observations indicated that the majority of these patients were either deceased or had been abandoned. Consequently, we performed a core outcome analysis on patients admitted to the ICU for less than 48 hours, and the results were consistent with the primary findings observed over the 28-day period. However, it is important to acknowledge that these findings are based on short-term follow-up and may not be applicable to follow-up periods extending to 60 days or longer.**

**Finally, the database is derived from studies conducted on U.S. populations, and the absence of external validation may limit the generalizability of the findings to patient populations in other countries or regions. Future research should focus on conducting high-quality prospective studies that incorporate external validation.**

- ****Reviewer’s Comment2:****

The discussion mentions that clinicians should monitor MCV, but it is unclear how this should influence clinical practice. Address whether MCV should be incorporated into sepsis risk stratification models or how it compares with other established biomarkers (e.g., lactate, SOFA score).

**Response:**
 We expanded the clinical implications in the Discussion.

**Revised Text (Lines 272-283):**
MCV may provide independent prognostic information from the perspectives of red blood cell metabolism and chronic inflammation. Previous studies have established that elevated lactate levels and increased SOFA scores are strong predictors of short-term mortality in critically ill patients^[32, 33]^. In this study, we found that MCV retains significant incremental predictive value even after adjusting for lactate. While the predictive capability of MCV alone may be less robust compared to that of lactate levels and SOFA scores, its low cost and ease of accessibility render it a valuable supplementary tool for risk stratification, particularly in resource-limited environments^[34]^. Drawing upon the findings of this study regarding the nonlinear association, we propose the inclusion of MCV as a supplementary indicator in the current sepsis risk stratification model. Specifically, for patients exhibiting an MCV greater than 83 fL, it is imperative for clinicians to exercise heightened vigilance and undertake a thorough evaluation that integrates lactate levels, inflammatory markers, and organ function scores.

We extend our thanks to the reviewers for their valuable feedback, which has significantly improved the quality of our manuscript.

Sincerely,
Hui-Zhen Tang, on behalf of all authors
